# The underestimated preventive effects of flexible sigmoidoscopy screening: re-analysis and meta-analysis of randomized trials

**DOI:** 10.1007/s10654-024-01120-w

**Published:** 2024-04-20

**Authors:** Hermann Brenner, Thomas Heisser, Rafael Cardoso, Michael Hoffmeister

**Affiliations:** 1 Division of Clinical Epidemiology and Aging Research, German Cancer Research Center (DKFZ), INF 581, Heidelberg, 69120 Germany; 2 German Cancer Consortium (DKTK), German Cancer Research Center (DKFZ), Heidelberg, Germany; 3 Medical Faculty Heidelberg, Heidelberg University, Heidelberg, Germany

**Keywords:** Colorectal cancer, Flexible sigmoidoscopy, Incidence, Risk, Screening, Trials

## Abstract

Flexible sigmoidoscopy (FS), which is less invasive, resource intensive and costly than colonoscopy, is among the recommended screening options for colorectal cancer (CRC). Four large randomized trials consistently reported statistically significant, albeit modest effects of screening by FS on CRC incidence. However, their effect estimates included cancers that were already prevalent at recruitment and could not have been prevented by screening. We performed a re-analysis and meta-analysis of two of the trials (including the largest one) to estimate reduction of truly incident cases by a single FS offered between 55 and 64 years of age among the “at risk study population” without prevalent CRC at recruitment. In meta-analyses of data reported after more than 15 years of follow-up, relative risk (95% CI) in intention-to-screen and per-protocol analyses were 0.71 (0.66–0.76) and 0.59 (0.55–0.65) for any CRC, and 0.52 (0.47–0.57) and 0.34 (0.30–0.39) for distal CRC, respectively. These results indicate much stronger effects than those suggested by the original reports and imply that a single screening FS can prevent approximately two out of three distal incident CRC cases within 15 + years of follow-up.

**Supplementary Information:**

The online version contains supplementary material available at 10.1007/s10654-024-01120-w.

## Introduction

Flexible sigmoidoscopy (FS) is among the recommended screening options for colorectal cancer (CRC) [1]. Nevertheless, it is not widely offered as primary screening examination even though its effectiveness in reducing CRC incidence and mortality has been demonstrated by randomized controlled trials (RCTs) [2–5], long before such evidence became available for screening colonoscopy [6]. From four large-scale RCTs, conducted in the United Kingdom (UK), Italy, Norway and the United States (US), data from more than 15 years of follow-up are meanwhile available [7–10]. In a recent pooled analysis of these RCTs, reduction of total and distal CRC incidence was estimated as 21% (95% CI 17–25%) and 32% (95% CI 27–37%), respectively, and the reduction of CRC mortality was estimated as 20% (95% CI 12–28%) in intention-to-screen analysis (no per-protocol analysis was performed) [11].

However, the incidence outcome in these effect estimates included cancers that were already prevalent but had yet remained undiagnosed at the time of recruitment. There is no way screening could have prevented these prevalent cancers, even though it could have led to their earlier detection. With respect to truly incident cases (i.e. cases that were not yet prevalent at recruitment), inclusion of prevalent cases violates a key principle of prevention trials that only people still at risk of developing the outcome one aims to prevent should be included. We have recently demonstrated based on data from the NordICC trial [6], the so far only randomised trial reporting on long-term effects of screening colonoscopy, that such “prevalence bias” may lead to strong underestimation of CRC screening effects [12].

The aim of this analysis was to estimate the effects of a single FS offered at ages 55–64 on total and distal CRC risk after excluding non-preventable cases that were prevalent at the time of recruitment from both the intervention group and the control group. Our analysis is based on data presented in four articles (two on the UK and two on the Italian trial) that were published after > 10 years and > 15 years of follow-up [2, 3, 7, 10]. Details on the trial designs and populations have been reported elsewhere and are summarised briefly in Table 1 and the Methods section. Both trials reported data from intention-to-screen analysis and per-protocol analysis in sufficient detail to enable deriving effect estimates for the prevention of truly incident CRC cases under plausible assumptions as outlined in the Methods section.

**Table 1:** Key characteristics and results on CRC incidence of the UK and Italian Flexible Sigmoidoscpy trials

Characteristic	UKFSST [2, 7]	Italian SCORE Trial [3, 10]
Target population	Average risk, 55–64 years	Average risk, 55–64 years
Screening Offer	Single FS	Single FS
Primary outcomes	CRC incidence and mortality	CRC incidence and mortality
Secondary endpoints	Proximal and distal CRC incidence, all-cause mortality, non-CRC mortality	Proximal and distal CRC incidence, advanced stage (UICC stage III and IV) CRC incidence, all-cause mortality, non-CRC mortality
Prescreening for eligibility / willingness to particpate	*N* = 368,142	*N* = 236,568
Included in Trial	*N* = 170,034	*N* = 34,296
Recruitment period	1994–1999	1995–1999
Control Group	*N* = 112,936	*N* = 17,148
Intervention Group	*N* = 57,098	*N* = 17,148
Screened	*N* = 40,621 (71%)	*N* = 9911 (58%)
1st report on CRC incidence [2, 3]	2010	2011
Median follow-up	11.2 years	10.5 years
2nd report on CRC incidence [7, 10]	2017	2022
Median follow-up	17.1 years	15.4 years

CI, confidence interval; CRC, colorectal cancer; FS, flexible sigmoidoscopy

We did not include data from the US and the Norwegian trials [4, 5, 8, 9], which did not report detailed results of per-protocol analyses, included different age ranges (55–74 and 50–64, respectively) and offered additional screening exams to all or part of the intervention group (a second FS after 3 or 5 years in the US trial, a fecal occult blood test in the Norwegian trial).

## Methods

### Study design and study populations

The UK Flexible Sigmoidoscopy Screening Trial (UKFSST), the largest of the four FS trials that published long-term effect estimates [2, 7], recruited 170,432 men and women in 14 centres across the UK (11 in England, 2 in Wales, 1 in Scotland) from November 1994 to March 1999. Participants aged 55–64 years with no history of CRC, adenomas or inflammatory bowel disease who had been sent a questionnaire to establish interest in screening beforehand were selected from 368,142 patients from 506 general practices. Participants were randomised in a 1:2 ratio to the intervention group (offered a single FS) and the control (usual-care) group. The final analysis included 170,034 participants: 57,098 participants in the intervention group of whom 40,621 (71%) were screened in hospital endoscopy clinics, and 112,936 participants in the control group (Table 1). Follow-up with respect to CRC incidence and mortality was performed by record linkage with registries. Primary outcomes were CRC incidence, including cases that were already prevalent at recruitment (and partly early detected by screening), and mortality. Secondary outcomes included incidence of proximal and distal CRC, all-cause mortality and mortality from causes other than CRC. Both intention-to-screen and per-protocol analyses were reported. The first report, which was based on follow-up data until December 2008, was published in April 2010 (median follow-up time: 11.2 years) [2], the second report included follow-up data until December 2014 (median follow-up time: 17.1 years) and was published in February 2017 [7].

The Italian Screening for Colon REctum (SCORE) trial followed a similar protocol as the UKFSST [3, 10]. Eligible and interested participants were identified through a questionnaire answered by 56,532 out of 236,568 men and women aged 55–64 who were randomly selected from six trial centers in Italy. A total of 34,296 eligible and interested participants (17,148 per group) were randomly assigned to the intervention group (which was invited to a single FS) and the control group between June 1995 and May 1999. The invitation was followed by 9911 participants (58%) in the intervention group. Follow-up with respect to CRC incidence and mortality was performed by regular record linkage of the trial database with regional hospital discharge records and the pathology department files in all geographic areas covered by the study, and by record linkage with regional mortality statistics at the end of the follow-up. Primary outcomes were incidence of CRC, including cases that were already prevalent at recruitment (and partly early detected by screening), and CRC-specific mortality. Secondary outcomes were incidence of proximal and distal CRC, incidence of advanced CRC (UICC stages III and IV), all-cause mortality and mortality not related to CRC. Results from both intention-to-screen and per-protocol analyses were reported. The first report, which was based on incidence and mortality follow-up data until December 2007 and 2008, respectively, was published in September 2011 (median follow-up time: 10.5 and 11.4 years for CRC incidence and mortality respectively) [3]. The second report, with CRC incidence data until 2012 (median follow-up time: 15.4 years) and CRC mortality data until 2014–2016 (median follow-up time 18.8 years), was published in 2022 [10].

### Statistical analysis

**Fig. 1:**
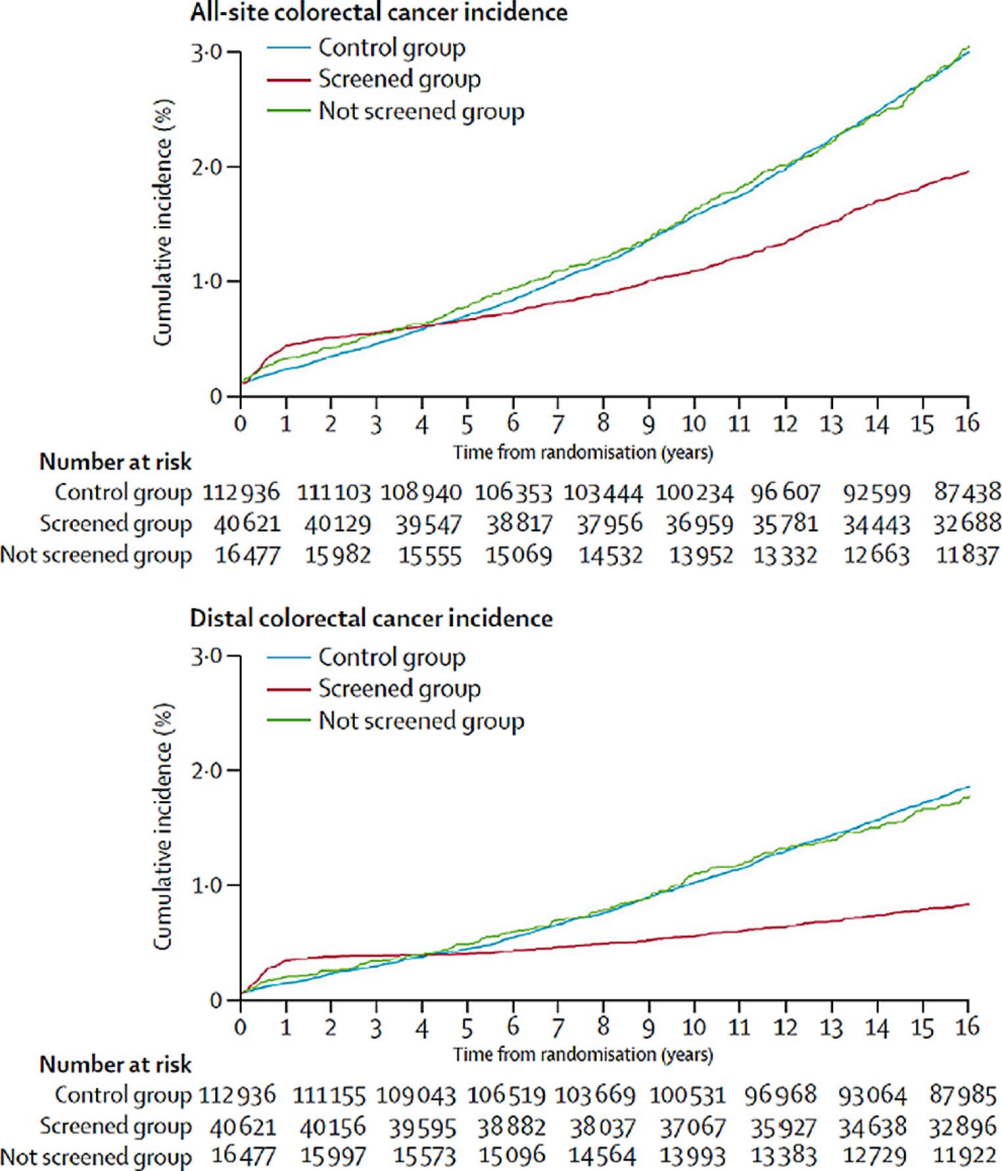
Reported cumulative colorectal cancer incidence among screened and not screened participants in the intervention group and participants in the control group in the United Kingdom Flexible Sigmoidoscopy trial. Edited extracts from Fig. 1 in the report from Atkin et al. [7]

The published data on CRC incidence from the trials included cases that were already prevalent at recruitment as well as truly incident cases diagnosed during follow up. We derived numbers of both prevalent and truly incident cases and estimated effectiveness of FS in reducing truly incident cases after excluding cases from the study population that were estimated to have been prevalent at recruitment in both the intervention and the control group. Our analyses are based on two key assumptions:

Firstly, prevalence of distal CRC at recruitment, which was estimated from distal CRCs detected by the FS and reported for screened participants only, was assumed to be the same in the intervention group and the control group (which should be ensured by the randomised study design and the large study populations), and furthermore the same among screened and unscreened participants in the intervention group. The latter is highly plausible for these two trials (but not necessarily so for other trials), given almost identical „cumulative incidence“ curves reported for unscreened participants in the intervention and control groups (see Fig. 1 in reference 7 and Fig. 2 in reference 10; edited extracts from the former are shown for illustration in Fig. 1).

Secondly, the ratio of total and distal CRC prevalence at recruitment was assumed to equal the ratio of total and distal CRC incidence in the absence of screening. This assumption implies comparable sojourn time in preclinical state for total and distal CRC. To account for uncertainties in this second assumption, we conducted sensitivity analyses assuming a 10% higher or 10% lower total-to-distal CRC prevalence ratio.

Based on these assumptions, the numbers of cases with prevalent distal CRC at recruitment among unscreened participants in the intervention group and the control group were derived as the product of the total numbers of participants in these groups and the detection rate of distal CRC among screened participants. Numbers of cases with prevalent CRC at recruitment at any site in each group was estimated by multiplying numbers of distal prevalent cases with the ratio of total to distal cases in the control group. The latter, which was unaffected by screening, were derived from reported trial data for the shorter follow-up period. Numbers of truly incident cases were derived by subtracting numbers of observed or estimated prevalent cases from the reported numbers of “incident” cases and used to estimate cumulative risk of truly incident total and distal CRC in each trial subgroup (control group, screened and unscreened participants in the intervention group). In addition, we derived relative risks of truly incident CRC (with 95% confidence interval, CI) for the intervention group compared to the control group (intention-to-screen analysis) and for the screened participants compared to the control group (per-protocol analysis). Equations for the calculations are provided in Supplementary Tables 1 and 2.

All analyses were carried out both for the interim results reported after a median of 11.2 or 10.5 years of follow-up, and for the final analyses reported after 17.1 or 15.4 years of follow-up in the UKFSST and the Italian SCORE trial, respectivey. In addition to calculations for the indvidual studies, we also carried out meta-analyses of relative risk estimates from both studies. Relative risk estimates and their 95% CIs were calculated according to Altman [13]. Meta-analyses using fixed effects models with inverse variance weighting were conducted using the R package ‘metafor’ R [14].

## Results

Table 2 shows the derivation of relative risk estimates of truly incident CRC at any site in both intention-to-screen and per-protocol analysis. All estimates are based on published data from the two trials as outlined in the methods section. Case numbers written in italic were not directly reported in the original publications, but were derived under plausible assumptions, as outlined in the methods section. Relative risk estimates for any CRC, including both prevalent cases at recruitment and truly incident CRC, that were derived from the reported count data (RR_any_) were identical or very close to the relative risk estimates that were reported in the articles (RR_rep_), which had been based on incidence rate data (using exact person-times) rather than count data. This also applies to the per-protocol estimates, even though our count-data based estimates were not adjusted for potential differences in users and nonusers of screening within the intervention groups, which seemed to be negligible in these two trials (as reflected in the almost identical “cumulative incidence” curves among unscreenied participants in the intervention group and the control group, illustrated in Fig. 1 for one of the trials).

**Table 2:** Relative risk estimates of incident CRC at any site as compared to relative risk estimates of any (prevalent or incident) CRC

Authors, year, country, follow-up	Group		Participants	Participants with CRC at any site^a^			Relative risk estimate (95% CI)			
				Any	Prevalent	Incident	Type of analysis	RR_rep_	RR_any_	RR_inc_
Atkin et al., 2010, UK, 11.2 years [2]	Control	Total	112,939	1818	*534*	*1284*		1.00 (Ref)	1.00 (Ref)	1.00 (Ref)
	Intervention	Total	57,099	706	*270*	*436*	Intention-to screen	0.77 (0.70–0.84)	0.77 (0.70–0.84)	0.67 (0.60–0.75)
		Screened	40,621	445	*192*	*253*	Per-protocol	0.67 (0.60–0.76)	0.68 (0.61–0.76)	0.55 (0.48–0.63)
Atkin et al., 2017, UK, 17.1 years [7]	Control	Total	112,936	3253	*534*	*2719*		1.00 (Ref)	1.00 (Ref)	1.00 (Ref)
	Intervention	Total	57,098	1230	*270*	*960*	Intention-to screen	0.74 (0.70–0.80)	0.75 (0.70–0.80)	0.70 (0.65–0.75)
		Screened	40,621	776	*192*	*584*	Per-protocol	0.65 (0.59–0.71)	0.66 (0.61–0.72)	0.60 (0.54–0.65)
Segnan et al., 2011, Italy, 10.5 years [3]	Control	Total	17,136	306	*128*	*178*		1.00 (Ref)	1.00 (Ref)	1.00 (Ref)
	Intervention	Total	17,136	251	*128*	*123*	Intention-to screen	0.82 (0.69–0.96)	0.82 (0.69–0.97)	0.69 (0.55–0.87)
		Screened	9,911	126	*74*	*52*	Per-protocol	0.69 (0.56–0.86)	0.71 (0.58–0.88)	0.50 (0.37–0.69)
Senore et al., 2022, Italy, 15.4 years [10]	Control	Total	17,136	468	*128*	*340*		1.00 (Ref)	1.00 (Ref)	1.00 (Ref)
	Intervention	Total	17,136	382	*128*	*254*	Intention-to screen	0.81 (0.71–0.93)	0.82 (0.71–0.94)	0.75 (0.63–0.88)
		Screened	9,911	184	*74*	*110*	Per-protocol	0.67 (0.56–0.81)	0.68 (0.57–0.81)	0.56 (0.45–0.69)

CI, confidence interval; CRC, colorectal cancer; Ref, reference; RR_rep_, reported relative risk of any CRC (derived from incidence rate data; including prevalent cases at recruitment); RR_any_, relative risk of any CRC (derived from count data; including prevalent cases at recruitment); RR_inc_, relative risk of truly incident CRC (derived from count data)

^a^Italic numbers were calculated from reported numbers as described in the methods section

However, substantially stronger risk reduction by FS was seen when prevalent CRC cases at recruitment that could not have been prevented by screening were excluded, i.e., when the analysis was focused on the potentially preventable truly incident cases (RR_inc_). Intention-to-screen estimates of RR_inc_ ranged from 0.67 to 0.75, compared to 0.74 to 0.82 for RR_rep_, meaning that the screening offer was associated with a 25 to 33% rather than a 18 to 26% reduction in risk of incident CRC. Even larger discrepancies were seen for relative risk estimates in the per-protocol analyses, with RR_inc_ ranging from 0.50 to 0.60, compared to a range of 0.65 to 0.69 for RR_rep_.

For distal CRC, RR_inc_ in intention-to-screen analyses ranged from 0.49 to 0.60, compared to RR_rep_ between 0.59 and 0.76 (Table 3). These results suggest risk reduction of truly incident distal CRC by the offer of FS screening to be in the order of 40 to 51% rather than 24 to 41%. Estimates of RR_inc_ in the per-protocol analyses ranged from 0.29 to 0.35, suggesting that use of FS reduced total risk of CRC by as much as 65–71% rather than by 40–56%, as suggested by the original reports.

**Table 3:** Relative risk estimates of incident distal CRC as compared to relative risk estimates of any (prevalent or incident) distal CRC

Authors, year, country, follow-up	Group		Participants	Participants with distal CRC^a^			Relative risk estimate (95% CI)			
				Any	Prevalent	Incident	Type of analysis	RR_rep_	RR_any_	RR_inc_
Atkin et al., 2010, UK, 11.2 years [2]	Control	Total	112,939	1192	*350*	*842*		1.00 (Ref)	1.00 (Ref)	1.00 (Ref)
	Intervention	Total	57,099	386	*177*	*209*	Intention-to screen	0.64 (0.57–0.72)	0.64 (0.57–0.72)	0.49 (0.42–0.57)
		Screened	40,621	215	126	*89*	Per-protocol	0.50 (0.42–0.59)	0.50 (0.43–0.58)	0.29 (0.24–0.37)
Atkin et al., 2017, UK, 17.1 years [7]	Control	Total	112,936	1987	*350*	*1637*		1.00 (Ref)	1.00 (Ref)	1.00 (Ref)
	Intervention	Total	57,098	592	*177*	*415*	Intention-to screen	0.59 (0.54–0.64)	0.59 (0.54–0.65)	0.50 (0.45–0.56)
		Screened	40,621	325	126	*199*	Per-protocol	0.44 (0.38–0.50)	0.45 (0.40–0.51)	0.34 (0.29–0.39)
Segnan et al., 2011, Italy, 10.5 years [3]	Control	Total	17,136	198	*83*	*115*		1.00 (Ref)	1.00 (Ref)	1.00 (Ref)
	Intervention	Total	17,136	152	*83*	*69*	Intention-to screen	0.76 (0.62–0.94)	0.77 (0.62–0.95)	0.60 (0.45–0.81)
		Screened	9,911	71	48	*23*	Per-protocol	0.60 (0.46–0.80)	0.62 (0.47–0.81)	0.35 (0.22–0.54)
Senore et al., 2022, Italy, 15.4 years [10]	Control	Total	17,136	297	*83*	*214*		1.00 (Ref)	1.00 (Ref)	1.00 (Ref)
	Intervention	Total	17,136	209	*83*	*126*	Intention-to screen	0.70 (0.59–0.84)	0.70 (0.59–0.84)	0.59 (0.47–0.73)
		Screened	9,911	89	48	*41*	Per-protocol	0.50 (0.39–0.63)	0.52 (0.41–0.66)	0.33 (0.24–0.46)

CI, confidence interval; CRC, colorectal cancer; Ref, reference; RR_rep_, reported relative risk of any distal CRC (derived from incidence rate data; including prevalent cases at recruitment); RR_any_, relative risk of any distal CRC (derived from count data; including prevalent cases at recruitment); RR_inc_, relative risk of truly incident distal CRC (derived from count data)

^a^Italic numbers were calculated from reported numbers as described in the methods section

Table 4 provides summary estimates of relative risk of truly incident CRC at any site and truly incident distal CRC for both studies combined. All of the effect estimates for truly incident CRC were substantially stronger than the effect estimates that were obtained when prevalent cases at recruitment were included. In none of the analyses for distal CRC did the 95% CIs of the relative risk estimates from both types of analyses overlap. Risk reduction of truly incident distal CRC by close to 50% in intention-to-screen analysis and by close to 70% in per-protocol analysis was consistently estimated both for the shorter and the longer follow-up period. In the most comprehensive analysis including more than 15 years of follow-up of participants from both studies, intention-to-screen and per-protocol analyses estimated offer and actual use of FS to reduce truly incident CRC at any site by 29% (95% CI 24–34%) and 41% (95% CI 35–45%), respectively. Incidence of distal CRC was estimated to be reduced by 48% (95% CI 43–53%) in intention-to-screen analysis and 66% (95% CI 61–70%) in per-protocol analysis.

**Table 4:** Meta-analyses of relative risk estimates for truly incident cases derived from both studies [2, 3, 7, 10]

Site	Follow-up [years]	Group		Relative risk estimate (95% CI)		
				Type of analysis	RR_any_ (95% CI)	RR_inc_ (95% CI)
Any	> 10 years UK: 11.2 Italy: 10.5	Control	Total		1.00 (Ref)	1.00 (Ref)
		Intervention	Total	Intention-to-screen	0.78 (0.72–0.85)	0.67 (0.61–0.74)
			Screened	Per-protocol	0.69 (0.62–0.76)	0.54 (0.48–0.61)
	> 15 years UK: 17.1 Italy: 15.4	Control	Total		1.00 (Ref)	1.00 (Ref)
		Intervention	Total	Intention-to-screen	0.76 (0.72–0.81)	0.71 (0.66–0.76)
			Screened	Per-protocol	0.66 (0.62–0.72)	0.59 (0.55–0.65)
Distal	> 10 years UK: 11.2 Italy: 10.5	Control	Total		1.00 (Ref)	1.00 (Ref)
		Intervention	Total	Intention-to-screen	0.67 (0.60–0.74)	0.51 (0.45–0.59)
			Screened	Per-protocol	0.53 (0.46–0.60)	0.30 (0.25–0.37)
	> 15 years UK: 17.1 Italy: 15.4	Control	Total		1.00 (Ref)	1.00 (Ref)
		Intervention	Total	Intention-to-screen	0.61 (0.56–0.66)	0.52 (0.47–0.57)
			Screened	Per-protocol	0.46 (0.42–0.52)	0.34 (0.30–0.39)

Ref, Reference; RR_any_, relative risk of any CRC (including prevalent cases at recruitment); RR_inc_, relative risk of truly incident CRC

## Discussion

In this article, we demonstrate that prevention of CRC, in particular prevention of distal CRC by a single FS offered at age 55–64 years is substantially stronger than suggested by published results of RCTs which included in their analyses CRC cases that were already prevalent at recruitment and therefore not preventable anymore. Our analyses suggest that people undergoing FS at around 60 years of age can reduce their risk to develop incident distal CRC within the following 15 years by approximately two thirds rather than approximately half as suggested by published results, which included prevalent cases at recruitment in their outcome measures. Whereas inclusion of prevalent CRCs at recruitment attenuated effect estimates of endoscopic screening on CRC incidence in conventional analysis, the earlier detection of such prevalent cases should rather be considered as an additional major asset of screening on top of a stronger than previously assumed preventive effect on CRC incidence [15].

Our analyses assumed equal prevalence at recruitment of distal CRC among participants in the intervention groups and the control groups and among screened and unscreened participants in the intervention groups. The former assumption is plausible due to the randomized study designs and the large sample size. The latter assumption is also plausible for the two trials included in our analysis, as published cumulative incidence curves were almost identical for unscreened participants in the intervention group and participants in the control group, indicating that use of the FS offer was not related to CRC risk in these two trials.

Nevertheless, it could be theoretically possible that not all distal CRCs that were prevalent at the time of recruitment became clinically manifest and diagnosed during the follow up in the unscreened participants in the intervention group and the control group. Even though their proportion would be expected to be very small, given the long follow-up period (> 15 years) and a mean sojourn time of preclinical CRC in the order of 3 to 6 years [16–18], the numbers and proportion of prevalent cancers at recruitment among all reported distal CRC could have been slightly smaller, and the numbers of truly incident cancers could have been slightly higher in these subgroups than assumed in our analyses. This would imply that underestimation of reduction of truly incident cases among screened participants (in whom prevalent cases at recruitment were disclosed by FS) may have been even stronger than suggested by our analysis.

In theory, screening might also also have led to some overdiagnoses of cases that would otherwise never have become diagnosed at lifetime. However, such overdiagnoses are expected to be rare for the age groups included in the trials [19]. Of greater concern may be imperfect sensitivity of FS to detect precursors of CRC and lack of re-screening which may account for the majority of the remaining truly incident cases.

For the effect estimates on total CRC incidence the additional assumption was made that the ratio of total and distal CRC prevalence at recruitment was the same as the ratio of the observed incidence total and distal CRC incidence in the absence of screening which implies that mean sojourn time in preclinical state would be the same for distal and proximal CRC. To account for potential variation in mean sojourn time according to cancer site, we conducted sensitivity analyses assuming 10% higher or lower total numbers of prevalent cancers at recruitment which yielded very similar results as the base case analyses (see Supplementary Table 3). Given that the majority of CRCs are located in the distal colon and rectum, such 10% differences in the overall number of prevalent cases at recruitment would reflect very large differences in the mean sojourn time of proximal and distal cancers. Even major variations in such sojourn times which appears unlikely would therefore have only a rather minor impact on our results.

We focused our analyses on RCT estimates of the effectiveness of FS. As previously demonstrated [12, 20–22], the concerns regarding underestimation of screening effects on CRC incidence by inclusion of non-preventable prevalent cancers at recruitment in the outcome measure similarly apply to the NordICC study, the so far only RCT reporting on long-term outcomes of screening colonoscopy [6]. In that trial, however, major differences in reported cumulative incidence of CRC between unscreened participants in the intervention group and participants in the control group suggest major selection effects in use of screening colonoscopy, making derivation of „prevalence-corrected“ effect estimates somewhat more complex. However, a recent modelling study suggested „prevalence-corrected“ effect estimates of screening colonoscopy on total CRC incidence to be very similar to the ones for distal CRC incidence derived for FS in this article [20].

Among the four large FS trials [2–5, 7–11], we chose the UKFSST [2, 7] and the Italian SCORE trial [3, 10] for demonstrating our point as these trials focused on the effects of a single screening FS at age 55–64 and reported the necessary data from both intention-to-screen and per-protocol analysis in detail, including cumulative incidence curves showing virtually identical cumulative incidence of unscreened participants in the intervention group and participants in the control group. The latter enabled straightforward derivation of “prevalence-bias corrected” effect estimates under plausible assumptions. Overall results of these two trials are largely comparable to results of the other two large FS trials from Norway and the US [4, 5, 8, 9], suggesting that the order of magnitude of incidence reduction by FS screening may be generalizable to other countries.

Our analyses focused on CRC incidence, one of the two primary outcomes of the FS screening trials for which underestimation of effects by inclusion of prevalent cases is relevant. We did not address effects on CRC mortality, the other primary outcome of the trials. In contrast to CRC incidence, there is no concern about including cases that were already prevalent at the time of recruitment in analyses for the mortality outcomes. In the contrary, screening by FS conveys its preventive effects on CRC mortality through both earlier detection of prevalent CRC cases and prevention of truly incident CRC cases [23, 24]. Interestingly, the “prevalence bias corrected estimates” of CRC incidence reduction derived in our analysis are very similar to the estimates of CRC mortality reduction reported from both trials. For example, for the > 17 year follow-up of the UKFSST, our “prevalence bias corrected estimates” of incidence reduction (intention-to-screen analysis: 30% for any CRC, 50% for distal CRC; per-protocol analysis: 40% for any CRC, 66% for distal CRC) almost perfectly match the estimates of CRC mortality reduction reported from that study (intention-to-screen analysis: 30% for any CRC, 46% for distal CRC; per-protocol analysis: 41% for any CRC, 66% for distal CRC).

Our findings of much stronger preventive effects of screening endoscopies with detection and removal of CRC precursors than those suggested by the published RCT results are in line with observations of strong decreases in CRC incidence in countries with widespread offers and use of screening colonoscopy, such as the US and Germany [25, 26]. For example, CRC incidence has almost halved in the last three decades in the US, where use of screening colonoscopy has become very common, with meanwhile more than 60% of people above 50 years of age having had a colonoscopy in the past 10 years [27]. This strong decline in incidence was achieved despite unfavourable trends in CRC risk factors, such as the increase in prevalence of obesity [28]. Furthermore, the decline was selectively seen in age groups with widespread use of screening colonoscopy, whereas CRC incidence was rising in younger age groups [26]. Although screening FS is expected to be slightly less effective than screening colonoscopy with respect to reduction of total CRC risk among those who use the screening offer, the necessary capacities and resources and high adherence rates may be easier to achieve for screening FS. Furthermore, combination of FS with screening by fecal immunochemical test could ensure early detection (or even prevention) of the vast majority of proximal cancers, in addition to the strong prevention of distal cancers [29, 30]. Along with possible extension of FS screening intervals from previously recommended 5 years to 10 years [31], this could make screnning FS a particularly effective and cost-effective CRC screening strategy that would be feasible even in countries lacking screening colonoscopy resources.

In summary, our analysis provides evidence for substantially stronger preventive effects of CRC screening by FS than those suggested by the published RCT results. It is plausible to assume that preventive effects of other CRC screening approaches have likewise been substantially underestimated by the type of prevalence bias addressed in our analysis. The substantially stronger than previously assumed preventive effects of CRC screening may have important implications on key parameters of CRC programs, such as cost-effectiveness and benefit-harm ratio, and may help to better define target populations, age range and intervals of recommended screening offers which should be carefully further evaluated by pertinent modeling studies. Most importantly, however, our results should encourage more widespread roll-out of CRC programs and use of CRC screening offers, which are among the most effective approaches in cancer prevention available to date [32].

### Electronic supplementary material

Below is the link to the electronic supplementary material.

Supplementary Material 1

## Abbreviations

CRC: colorectal cancer
FS: flexible sigmoidoscopy
NordICC: Nordic-European Initiative on Colorectal Cancer
RCT: randomized controlled trial
RR^any^: relative risk of colorectal cancer (including prevalent cases), derived from reported count data
RR^inc^: relative risk of incident colorectal cancer, derived from reported count data
RR^rep^: reported relative risk of colorectal cancer (including prevalent cases)
SCORE: Screening for Colon REctum
UK: United Kingdom
UKFSST: United Kingdom Flexible Sigmoidoscopy Screening Trial
US: United States
