# Supplementary material for: The underestimated preventive effects of flexible sigmoidoscopy screening: re-analysis and meta-analysis of randomized trials
Source: Eur J Epidemiol. 2024 Apr 20;39(7):743–51. doi: 10.1007/s10654-024-01120-w (PMC11343976; doi:10.1007/s10654-024-01120-w)
Supplement: Supplementary file 1 — Supplementary Material 1 [file 10654_2024_1120_MOESM1_ESM.docx]

**Supplementary Table 1.** Calculation of relative risk estimates of incident distal CRC.^a^

| Group | | Partici-pants | Participants with distal CRC^a^ | | | Relative risk estimate (95% CI) | |
| --- | --- | --- | --- | --- | --- | --- | --- |
|  |  |  | Any | Prevalent  at recruitment | Incident | Type of analysis | RR_inc_ |
|  |  |  |  |  |  |  |  |
| Control | Total | **N_0_** | **A_D0_** | P_D0_=N_0_×P_D11_/N_11_ | I_D0_=A_D0_-P_D0_ |  | 1.00 (Ref) |
| Inter-vention | Total | **N_1_** | **A_D1_** | P_D1_=N_1_×P_D11_/N_11_ | I_D1_=A_D1_-P_D1_ | Intention-to-screen | (I_D1_/(N_1_-P_D1_))/(I_D0_/(N_0_-P_D0_)) |
|  | Screened | **N_11_** | **A_D11_** | **P_D11_** | I_D11_=A_D11_-P_D11_ | Per-protocol | (I_D11_/(N_11_-P_D11_))/(I_D0_/(N_0_-P_D0_)) |

CI, confidence interval; CRC, colorectal cancer; Ref., reference; RR_inc_, relative risk of truly incident CRC

^a^ bold numbers indicate reported numbers from which the other numbers are derived.

**Supplementary Table 2.** Calculation of relative risk estimates of CRC at any site^a^

| Group | | Partici-pants | Participants with CRC at any site^a^ | | | Relative risk estimate (95% CI) | |
| --- | --- | --- | --- | --- | --- | --- | --- |
|  |  |  | Any | Prevalent  at recruitment | Incident | Type of analysis | RR_inc_ |
|  |  |  |  |  |  |  |  |
| Control | Total | **N_0_** | **A_A0_** | P_A0_=P_D0_^b^ | I_A0_=A_A0_-P_A0_ |  | 1.00 (Ref) |
| Intervention | Total | **N_1_** | **A_A1_** | P_A1_=P_D1_×(A_A0_/A_D0_)^b^ | I_A1_=A_A1_-P_A1_ | Intention-to screen | (I_A1_/(N_1_-P_A1_))/(I_A0_/(N_0_-P_A0_)) |
|  | Screened | **N_11_** | **A_A11_** | P_A11_=P_D11_×(A_A0_/A_D0_)^b^ | I_A11_=A_A11_-P_A11_ | Per-protocol | (I_A11_/(N_11_-P_A11_))/(I_A0_/(N_0_-P_A0_)) |

CI, confidence interval; CRC, colorectal cancer; Ref., reference; RR_inc_, relative risk of truly incident CRC

^a^ bold numbers in Supplementary Tables 1 and 2 indicate reported numbers from which the other numbers are derived

^b^ ratios (A_A0_/A_D0_) derived from the publications with the shorter follow-up period

**Supplementary Table 3.** Relative risk estimates of truly incident CRC at any site as compared to relative risk estimates of reported any (prevalent or incident) CRC: sensitivity analyses

| Authors,  year, country,  follow-up |  | Relative risk estimate (95% CI) | | | |
| --- | --- | --- | --- | --- | --- |
|  | Type of analysis | RR_rep_ | RR_inc,bc_ | RR_inc,-10%_ | RR_inc,+10%_ |
|  |  |  |  |  |  |
| Atkin et al,  2010, UK,  11.2 years [2] |  | 1.00 (Ref) | 1.00 (Ref) | 1.00 (Ref) | 1.00 (Ref) |
|  | Intention-to screen | 0.77 (0.70-0.84) | 0.67 (0.60-0.75) | 0.68 (0.62-0.76) | 0.66 (0.59-0.74) |
|  | Per-protocol | 0.67 (0.60-0.76) | 0.55 (0.48-0.63) | 0.57 (0.50-0.64) | 0.53 (0.46-0.61) |
|  |  |  |  |  |  |
| Atkin et al, 2017, UK,  17.1 years [7] |  | 1.00 (Ref) | 1.00 (Ref) | 1.00 (Ref) | 1.00 (Ref) |
|  | Intention-to screen | 0.74 (0.70-0.80) | 0.70 (0.65-0.75) | 0.70 (0.65-0.76) | 0.69 (0.64-0.75) |
|  | Per-protocol | 0.65 (0.59-0.71) | 0.60 (0.54-0.65) | 0.60 (0.55-0.66) | 0.59 (0.54-0.65) |
|  |  |  |  |  |  |
| Segnan et al, 2011, Italy,  10.5 years [3] |  | 1.00 (Ref) | 1.00 (Ref) | 1.00 (Ref) | 1.00 (Ref) |
|  | Intention-to screen | 0.82 (0.69-0.96) | 0.69 (0.55-0.87) | 0.71 (0.57-0.89) | 0.67 (0.52-0.85) |
|  | Per-protocol | 0.69 (0.56-0.86) | 0.50 (0.37-0.69) | 0.54 (0.40-0.72) | 0.47 (0.33-0.65) |
|  |  |  |  |  |  |
| Senore et al, 2022, Italy,  15.4 years [10] |  | 1.00 (Ref) | 1.00 (Ref) | 1.00 (Ref) | 1.00 (Ref) |
|  | Intention-to screen | 0.81 (0.71-0.93) | 0.75 (0.63-0.88) | 0.76 (0.64-0.89) | 0.74 (0.62-0.87) |
|  | Per-protocol | 0.67 (0.56-0.81) | 0.56 (0.45-0.69) | 0.57 (0.47-0.71) | 0.54 (0.43-0.68) |

CI, confidence interval; CRC, colorectal cancer; Ref., reference; RR_rep_, reported relative risk of any CRC (including prevalent cases);

RR_inc,bc_, relative risk of truly incident CRC (base case analysis);

RR_inc,-10%_, relative risk of truly incident CRC (sensitivity analysis assuming 10% lower prevalence of CRC at any site);

RR_inc,+10%_, relative risk of truly incident CRC (base case analysis assuming 10% higher prevalence of CRC at any site)
